# Facile preparation of cobalt-oxide nanostructures with enriched cobalt(ii) ion surface using *Solanum melongena* for energy application†

**DOI:** 10.1039/d5ra02960k

**Published:** 2025-09-02

**Authors:** Abdul Jaleel Laghari, Umair Aftab, Muhammad Ishaque Abro, Aneela Tahira, Elmuez Dawi, Muhammad Ali Bhatti, Antonia Infantes-Molina, Melanie Emo, Brigitte Vigolo, Rafat M. Ibrahim, Zafar Hussain Ibupoto

**Affiliations:** a Department of Metallurgy and Materials, Mehran University of Engineering and Technology 76080 Jamshoro Sindh Pakistan ishaque.abro@faculty.muet.edu.pk; b Institute of Chemistry, University of Sindh Jamshoro Sindh 76080 Pakistan zaffar.ibhupoto@usindh.edu.pk; c Université de Lorraine, CNRS, IJL F-54000 Nancy France; d Department of Inorganic Chemistry, Crystallography and Mineralogy, Unidad Asociada al ICP-CSIC, Faculty of Sciences, University of Malaga Campus de Teatinos Malaga 29071 Spain; e Centre for Environmental Sciences, University of Sindh Jamshoro Sindh 76080 Pakistan; f Institute of Chemistry, Shah Abdul Latif University Khairpur Mirs Sindh Pakistan; g College of Humanities and Sciences, Department of Mathematics and Sciences, Ajman University P. O. Box 346 Ajman United Arab Emirates; h Physics Department, Faculty of Science, Taibah University Al-Madaina Al Munawarah 42353 Saudi Arabia

## Abstract

The increasing demand for efficient energy conversion and storage systems necessitates the development of high-performance, cost-effective electrode materials. To address this challenge, we employed rotten *Solanum melongena* (eggplant) juice as a precursor for the fabrication of low-cost, earth-abundant, and active electrode materials based on cobalt oxide (Co_3_O_4_) nanostructures. Different volumes of rotten *Solanum melongena* juice (5 mL, 10 mL, 15 mL, and 20 mL) were utilized during the precipitation process to synthesize Co_3_O_4_ nanostructures. These nanostructures were characterized in terms of crystal quality, surface morphology, surface chemical composition, and electrochemical properties. Notably, the synthesis using 15 mL of rotten *Solanum melongena* juice resulted in a highly efficient electrode material for oxygen evolution reaction (OER) in 1 M KOH electrolytic solution, with an overpotential of 276 mV at 10 mA cm^−2^. In addition to its electrocatalytic properties, the Co_3_O_4_ electrode material was evaluated for supercapacitor applications, demonstrating a specific capacitance of 1303.13 F g^−1^, an energy density of 28.96 W h kg^−1^ at 1.25 A g^−1^, and a 97% specific capacitance retention over 30 000 galvanic charge–discharge cycles.

## Abstract 2

The *Solanum melongena* juice shows the contributing role towards enhanced electrochemical performance of Co_3_O_4_ nanostructures during supercapacitor and OER applications.

## Graphical abstract

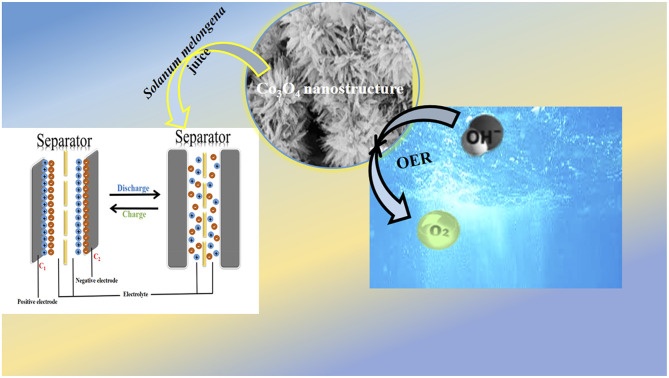

## 1 Introduction

A considerable amount of attention has been focused on renewable energy generation and energy storage to fulfill the global energy demand in the most efficient manner.^1–9^ The current global situation is more concentrated on the remedy of environmental pollution and exhaustive aspects of fossil fuels; therefore it has been deeply realised to fabricate the sustainable energy conversion and storage systems to mitigate the energy shortage.^1,3,6^ The supercapacitors are making the energy storage devices and have received a lot of focus in recent times.^6^ The supercapacitors have been associated with high power density, long cycling operational time, and swift charge–discharge capacity compared to Li-ion batteries.^6^ But the supercapacitors suffer from poor energy density, which is the main barrier to their practical use. The electrocatalytic water splitting is one of the potential energy conversion systems and has been known as an efficient technology to develop a sustainable hydrogen generation source.^7,8^ The oxygen evolution reaction (OER) has a great impact on the performance of electrocatalytic water splitting; however, OER exhibits slow kinetics, thus resulting in large overpotentials.^9^ The slow kinetics and high energy barrier of OER strongly limit the practical use of electrocatalytic water splitting for the efficient hydrogen generation.^9^

Properties like high catalytic activity, better electrical conductivity, sufficient surface area and porosity, good stability/durability and superior specific capacitance are required for the electrode material to perform well for water splitting and supercapacitor application.^10–18^ Transition metals such as nickel, iron, zinc, and cobalt based oxides are highly promising and potential substitutes for the noble metal-based electrocatalysts in electrochemical water splitting and supercapacitor application.^19–22^ Compared to CoO and Co_2_O_3_, Co_3_O_4_ is the most stable phase of cobalt oxide.^23,24^ The Co_3_O_4_ based materials have been synthesized by variety of potential methods such as combustion process,^25^ hydrothermal,^26^ Pechiney,^27^ co-precipitation,^28^ green approach,^29^ electrospinning method,^30^ solvothermal,^31^ and sol gel process.^32^ Green preparation of Co_3_O_4_ has demonstrated promising results in terms of influencing its surface properties, morphology, and stoichiometric variation. As a result, the electrochemical performance of Co_3_O_4_ is improved.^33,34^ By utilizing green synthesis, a variety of natural reducing, capping, and stabilizing agents can be utilized to enhance the surface-active sites, charge transport, highly compatible surfaces, tunable electron transport pathways, and the stability of materials against harsh electrolytes.^35,36^ Thus, different plant extracts have been used to synthesize a wide range of metal oxides nanostructures (ZnO, Fe_2_O_3_, AgO, CuO, Al_2_O_3_*etc.*).^37^ Previously, *Moringa oleifera* extract was used to synthesize Co_3_O_4_, and the results were highly promising in the development of electrode materials for supercapacitors.^38^ As a template, *Solanum tuberosum* leaf extract was also used to prepare Co_3_O_4_ nanostructures, and the electrochemical and antibacterial properties of Co_3_O_4_ have been greatly enhanced as a result.^39^ It has been discovered that several natural organic compounds act as catalysts during oxidation and reduction reactions as well as accelerating electron transport during oxidation of water.^40^ The *Solanum melongena* plant contains a variety of phenolic compounds and anthocyanins that possess reducing and oxidizing properties.^41,42^ To enhance the electrochemical performance of Co_3_O_4_ nanostructures, the hypothesis presented here proposes that rotten *Solanum melongena* extract can be used to tailor the sizes, shapes, and chemical compositions of the nanostructures. This could be possible through the exploitation of different phytochemicals of the *Solanum melongena* extract owing to their reducing, capping, stabilizing, and structure-directing agent properties. These characteristics of *Solanum melongena* extract during the synthesis of Co_3_O_4_ have not been investigated in the past; hence, this is the first kind of study. Therefore, the Co_3_O_4_ based material could be incorporated into the development of high-performance electrode materials for energy conversion and storage through the combined effect of various phytochemicals in *Solanum melongena*. As a result of the variable properties of cobalt-based materials, OER half-cell water splitting may be enabled at a low overpotential due to the use of rotten *Solanum melongena* juice. In light of these circumstances, the present study was carried out to synthesize Co_3_O_4_ based materials using different amounts of *Solanum melongena* juice, and it has been successfully applied to the development of an electrode material for use in OER and supercapacitors.

## 2 Materials and methods

### 2.1 Used chemicals

Cobalt chloride (CoCl_2_·6H_2_O), urea (CH_4_N_2_O), 85% potassium hydroxide, silicon paper, alumina (Al_2_O_3_) 0.03 μm slurry, nickel foam, and 20% ethanol (C_2_H_5_OH) were purchased from Sigma-Aldrich in Karachi Sindh Pakistan. The electrolyte solution and growth of materials were prepared using deionized water. The vegetable, *Solanum melongena*, was purchased from a local market in Jamshoro, Sindh.

### 2.2 Green mediate preparation of Co_3_O_4_ based materials using rotten *Solanum melongena* juice during precipitation method

A rotten *Solanum melongena* sample was collected from a local market in Jamshoro and washed several times with deionized water prior to being used to synthesize the Co_3_O_4_ nanostructure. To prepare the juice, rotten *Solanum melongena* (250 gm) was put into the domestic juicer machine and filtered using filter paper. We collected approximately 150 mL of *Solanum melongena* juice and stored it at 4 °C in a refrigerator. Afterwards, Co_3_O_4_ nanostructures were prepared using the precipitation method with and without rotten *Solanum melongena* juice. A variety of quantities of *Solanum melongena* juice were used in order to better understand the effects of rotten *Solanum melongena* juice on the structure and functionality of Co_3_O_4_. As a natural source of reducing, capping, and stabilizing agents, the juice of rotten *Solanum melongena*s can result in variation in shape orientation, stoichiometric composition, and size of Co_3_O_4_ nanostructures. In a typical preparation, 250 mL of deionized water contains 0.1 M cobalt chloride hexahydrate (5.92 gm) and 0.1 M urea (1.5 gm). Five growth solutions were prepared, four of which contained rotten *Solanum melongena* juice in amounts of 5 mL, 10 mL, 15 mL and 20 mL, as well as 0.1 M cobalt chloride hexahydrate (5.92 gm) and 0.1 M urea (1.5 gm). In addition, the fifth growth solution was prepared without rotten *Solanum melongena* juice and contained only 0.1 M cobalt chloride hexahydrate and 0.1 M urea, as well as a label indicating that it was pure cobalt oxide. In the following steps, five beakers containing growth solutions were covered with aluminum sheets and placed in an electric oven preheated to 95 °C for five hours. After collecting the cobalt hydroxide product, it was washed several times with ethanol and deionized water, and then dried overnight at 60 °C. Following this, the product was thermally annealed for three hours at 500 °C. The heating rate of the furnace was 8 °C min^−1^ in the presence of an air atmosphere. Therefore, Co_3_O_4_ nanostructures obtained with and without rotten *Solanum melongena* juice were characterized structurally and electrochemically. Co_3_O_4_ samples prepared from *Solanum melongena* juice are referred to as cobalt oxide-*Solanum melongenas* (CE) in the manuscript. ESI Scheme 1† illustrates the synthesis and interaction with the heterostructures.

### 2.3 The structure and compositional studies of green mediated Co_3_O_4_ nanostructure

The morphology of Co_3_O_4_ nanostructures was investigated through scanning electron microscopy (SEM) using a JEOL JSM-6480A operating at 20 kV. Additional structural analyses were conducted *via* transmission electron microscopy (TEM) and high-resolution transmission electron microscopy (HRTEM) using a JEOL JEM-ARM 200F Cold FEG microscope, operating at 200 kV and equipped with a probe corrector (*C*_s_). X-ray diffraction (XRD) patterns, obtained using a Philips PANalytical diffractometer with CuKα radiation (*λ* = 1.5418 Å) at 45 kV and 45 mA, were analyzed *via* HighScore Plus software to assess purity and crystallinity.^42^ Chemical states and surface composition were examined through X-ray photoelectron spectroscopy (XPS) using a monochromatic Al Kα source (1486 eV) under ultra-high vacuum (10^−10^ mbar), with results analyzed at a resolution of 0.651 eV on the 7/2 line of full width at half maximum. Atomic structure was further analyzed using a JEOL JEM ARM 200F Cold FEG microscope equipped with spherical aberration (*C*_s_). A phytochemical analysis of *Solanum melongena* juice was conducted at the University of Sindh, Jamshoro, employing various tests, including iodine, alkaloids, Molisch, Fehling's, Benedict's, Barfoed's, Dragendorff's, Mayer's, Hager's, Wagner's, and tannic acid tests. Fourier-transform infrared (FTIR) spectroscopy was performed using a PerkinElmer Spectrum Two instrument across the range of 4000 to 400 cm^−1^.

### 2.4 The fabrication of green mediated Co_3_O_4_ nanostructures-based electrode material for the energy conversion and storage applications

All electrochemical tests for OER characterization were conducted using a VERSASTAT 4-500 analytical potentiostat with various electrochemical modes, including linear sweep voltammetry (LSV), cyclic voltammetry (CV), electrochemical impedance spectroscopy (EIS), and chronopotentiometry in 1.0 M KOH aqueous solution. An electrochemical cell system with three electrodes was employed. A silver–silver chloride electrode (Ag/AgCl) filled with 3 M potassium chloride (KCl) electrolyte served as the reference electrode, a glassy carbon electrode (GCE) as the working electrode, and a graphite rod as the counter electrode. The catalytic material ink was prepared by mixing 10 mg of each Co_3_O_4_ nanostructure sample with 5 mL of deionized water and 0.3 mL of Nafion (5%) as a binder. The GCE had a diameter of approximately 3 mm (area: 0.071 cm^2^). Catalyst ink (5 μL, loading mass: 0.02 mg) was sprayed onto the GCE and dried by blowing air. CV and LSV tests were conducted on 1 M KOH electrolyte to characterize the OER. Prior to LSV, CV was used at a scan rate of 10 mV s^−1^ to stabilize the electrode. The reference electrode experimental potential was converted to the reversible hydrogen electrode potential using the Nernst equation. Electrochemical impedance spectroscopy (EIS) was utilized to measure charge transfer resistance under conditions of 100 kHz to 0.1 Hz, with sinusoidal potentials of 5 mV and 1.4 V *vs.* RHE as OER onset potentials.

Z-view software was used to simulate the EIS data to determine the appropriate electric circuit for accurate estimation of charge transfer resistance. A durability experiment was then conducted at current densities of 20 and 40 mA cm^−2^ for 15 and 30 hours, respectively. To calculate the electrochemical active surface area (ECSA), CV curves were measured at various scan rates in the non-Faradic region. For the conversion of experimental potentials measured against Ag/AgCl and the calculation of Tafel slopes, the following mathematical relations were applied.1
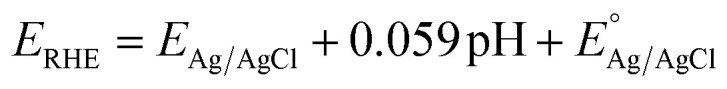
whereas, 
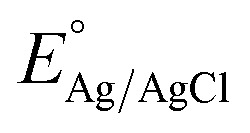
 is 0.2412

2 Overpotential (*η*) = Onset potential (*E*_RHE_)(V) − 1.23 (V)

Tafel equation was applied to LSV curves for the calculation of Tafel Slopes.

3 *η* = *b* log *j* + *a*

Herein, *b* is Tafel slope, *η* overpotential and *j* is current density.

An electrode material based on Co_3_O_4_ nanostructure was also studied for electrochemical supercapacitor applications by dip coating Co_3_O_4_ nanostructure on nickel foam. The purpose of using nickel as an electrode substrate was to deposit electrode materials due to its high surface area and the possibility of depositing large amounts of material. A nickel foam sample measuring 2 cm^2^ was dipped into Co_3_O_4_ ink that contained 5% Nafion as a binder, dried for five minutes, and then repeated several times. Material adhesion was enhanced by sintering at 65 °C for one hour at low temperature. It was found that around 2 mg of electrode material was loaded onto the nickel foam. A Co_3_O_4_ nanostructure was deposited on nickel foam and used as a working electrode, a graphite rod as a counter electrode and (Ag/AgCl, filled with 3 M KCl) as a counter electrode and a reference electrode. To characterize Co_3_O_4_ nanostructures prepared in 3.0 M KOH, galvanostatic charge–discharge (GCD) and cyclic voltammetry (CV) were used. The concentration of 3.0 M KOH was chosen due to the fact that high amounts of hydroxide ions can be adsorption onto the electrode material in order to demonstrate the capacity of the material to store charge efficiently. Using repeatable GCD cycles, the percent specific capacitance retention and coulombic efficiency of electrode materials were calculated for 30 000 cycles to ensure durability and cyclic stability.^43^ The formulae for specific capacitance retention and coulombic efficiency are given in eqn (S1) and (S2).†

## 3 Results and discussion

### 3.1 Morphology, crystal quality and chemical composition characterization of as prepared Co_3_O_4_ materials

**Fig. 1:**
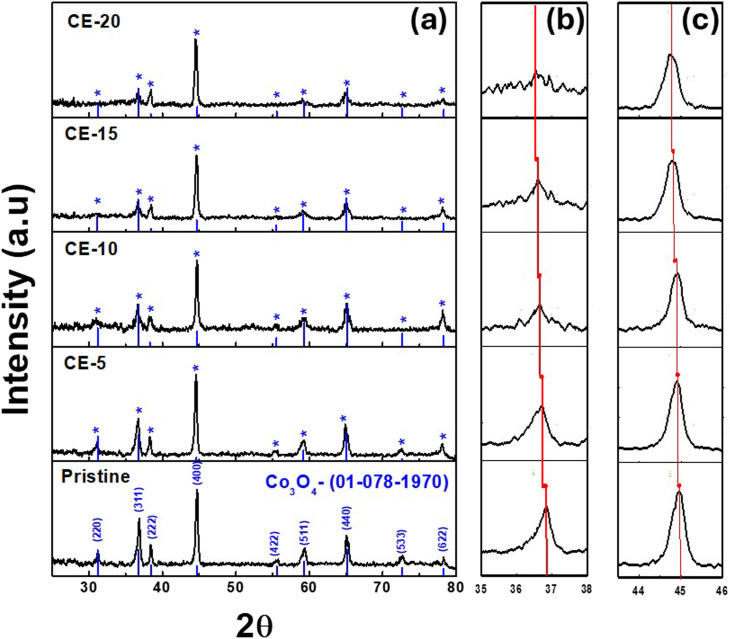
(a) XRD patterns of Co_3_O_4_ synthetized without rotten *Solanum melongena* juice (pristine) and with 5 mL (CE-5), 10 mL (CE-10), 15 mL (CE-15) and 20 mL (CE-20) of rotten *Solanum melongena*s juice. (b) (311) plane peak shift of corresponding XRD patterns, (c) (400) plane main peak shift of corresponding XRD patterns.

Using XRD analysis, we determined the phase and purity of pure Co_3_O_4_ and Co_3_O_4_ samples prepared with rotten *Solanum melongena* juice. Fig. 1 illustrates the measured reflections of each sample. The diffraction peaks were observed to be located at two-theta angles starting at 31.40°, 37.02°, 38.80°, 44.95°, 55.61°, 59.46°, 65.32°, 77.21°, and 79.11°, and were labeled as corresponding diffraction patterns (220), (311), (222), (422), (511), (440), (322), and (622). The measured diffraction patterns correspond to a cubic phase of Co_3_O_4_ nanostructure, according to the standard JCPDS (card no: 01-078-1970).

Using rotten *Solanum melongena* juice provided redox compounds that affected the relative intensity of Co_3_O_4_ peaks, resulting in slight differences between peak intensities using 5 mL (EC-5), 10 mL (EC-10), 15 mL (EC-15), and 20 mL (EC-20). According to the XRD analysis, the *Solanum melongena*-mediated preparation of Co_3_O_4_ was successful in the presence of *Solanum melongena* juice. *Solanum melongena* juice did not alter the Co_3_O_4_ phase, even at high concentrations, as compared to the synthesis process occurring without *Solanum melongena* juice (pristine Co_3_O_4_).

The magnified image (Fig. 1) of the (311) and (400) planes reveal that the peak shifts to the left side as a result of the compressing effect induced by the phytochemicals in *Solanum melongena* juice. With increasing amounts of *Solanum melongena* juice, the intensity of the peaks decreased, indicating a decrease in the crystallinity of Co_3_O_4_. The samples were found to be extremely pure and did not contain any other impurities. Information extracted from the XRD data is provided in ESI Table (S1),† further confirms that the crystallinity index decreases while *d*-spacing and lattice parameter are increases by the addition of rotten *Solanum melongena* during synthesis.

Table 1 lists the phytochemicals identified during the phytochemical analysis. These components of *Solanum melongena* juice play a dynamic role in affecting the structural, size, and surface properties of Co_3_O_4_ nanostructures. Phytochemical analysis revealed that phenolic compounds, alkaloids, tannins, and flavonoids can act as capping, reducing, stabilizing, and surface-modifying agents.

Phytochemical analysis of rotten *Solanum melongena* juice^a^

**Table:** 

Flavonols	Flavones	Flavonoids	Phenolic acid	Alkaloids
+	+	+	+	+

a + Present.

**Table:** 

Tannins	Saponins	Reducing sugar	Non-reducing sugar	Ascorbic acid
+	+	+	+	+

The method used for the phytochemical analysis is given in the ESI Section.†

**Fig. 2:**
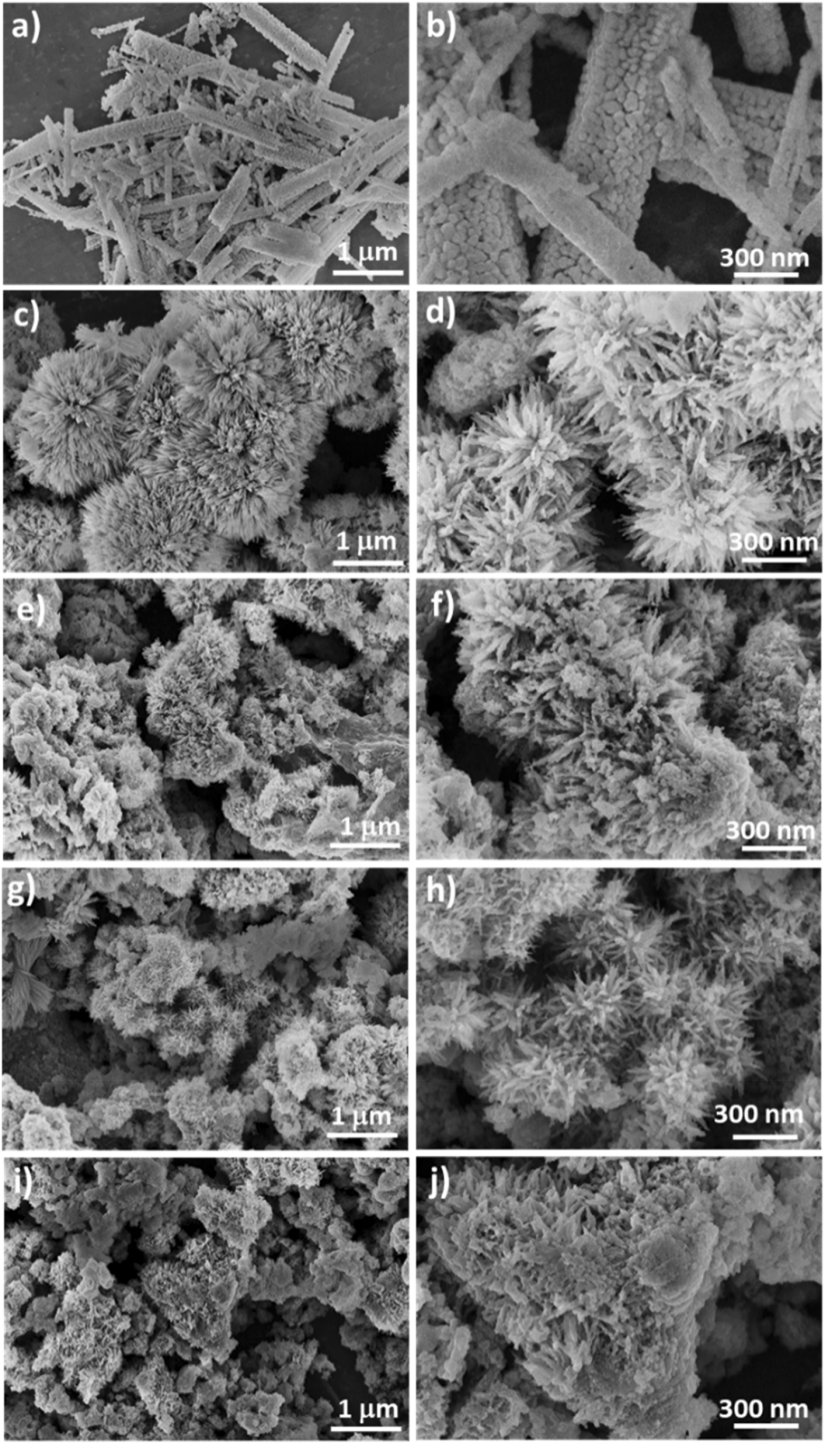
SEM images at different magnifications of Co_3_O_4_ synthetized (a and b) without rotten *Solanum melongena* juice and (c and d) with 5 mL, (e and f) 10 mL, (g and h) 15 mL and (i and j) 20 mL of rotten *Solanum melongena* juice.

Fig. 2 depicts typical SEM images of pure Co_3_O_4_ and Co_3_O_4_ nanostructures mediated by *Solanum melongena* at different magnifications. The Co_3_O_4_ is typically arranged in an elongated rod-like morphology composed of assembled nanoparticles glued together, with a length of a few microns and a diameter of 10–150 nm (Fig. 2a and b). It has been observed that in all cases where *Solanum melongena* juice is used in the interim, Co_3_O_4_ nanostructures mediated by *Solanum melongena* appear as urchin-like structures with needles no larger than 10 nanometers in diameter. According to (Fig. 2c–j), these nanostructures will exhibit a superior surface compared to the thick pure Co_3_O_4_ material, which has a thin surface due to the self-assembling of nanoparticles into defected nanorods. When the rotten *Solanum melongena* juice amount was increased from 5 mL, 10 mL, 15 mL during the synthesis process, an effect without a rigorous tendency was observed (Fig. 2c–h). It appears that the size of the urchins decreases from 500 nm^−1^ nm for 5 mL and 10 mL to a majority of urchins falling below 300–500 nm for Co_3_O_4_ prepared with 15 mL of rotten *Solanum melongena* juice. Additionally, some isolated particles are dispersed within the urchins. In the case of the highest quantity of *Solanum melongena* juice, 20 mL, well-defined nanostructures became less difficult to detect and aggregated into a denser structure of *Solanum melongena* juice, with variable surface morphology. As shown in Fig. 2, the largest amount of rotten *Solanum melongena* juice (Fig. 2i and j) has completely changed its morphology into nanosheets decorated with nanoparticles.

As can be seen in Fig. 2, 5 mL, 10 mL, and 15 mL of rotten *Solanum melongena* juice summarized the thin nanorod into a flower shape. The transformation of morphology may be related to the use of phytochemicals from the juice of *Solanum melongena* during the growth process. Since phytochemicals, particularly phenolic compounds, have reducing properties and possess hydroxyl terminated groups in cyclic rings. The two aspects of *Solanum melongena* juice phytochemicals influenced the reaction growth rate, resulting in a change in shape orientation from a rod to an urchin. *Solanum melongena* juice contains a wide range of phytochemicals with redox properties and also reducing properties for the surface modification of nanomaterials, in terms of size and shape variation. We propose that phytochemicals, particularly phenolic substances and reducing sugars, may play a role during the growth process by interfering with the Co ions by changing the growth kinetics and nucleation rate. According to the SEM studies shown in Fig. 2, the overall morphology and size of nanostructures were significantly altered. In connection with the XRD studies, it has been shown that the two theta angle was slightly changed, which may have been as a consequence of phytochemicals altering the nucleation rate for crystal growth during synthesis. As shown in Fig. 1, this shift in two-theta angle was observed.

SEM analysis revealed that *Solanum melongena* juice's oxidation and reductive compounds were the main mediators and responsible for tuning the morphology of Co_3_O_4_ nanostructures. As a result of its ability to tailor surface and electron communication, *Solanum melongena* juice could play a significant role in the final application of Co_3_O_4_ nanostructures.^38,39^

**Fig. 3:**
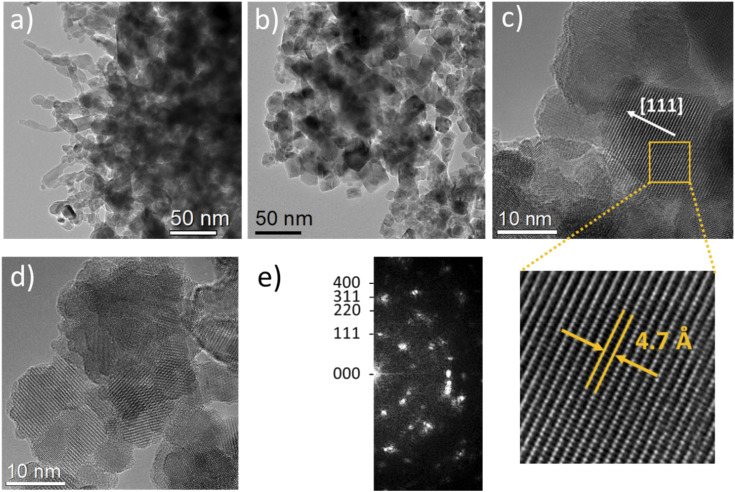
(a and b) TEM and (c and d) HRTEM micrographs at different magnifications of Co_3_O_4_ sample prepared with 15 mL of rotten *Solanum melongena* juice and (e) corresponding fast Fourier transform of (d).

TEM/HRTEM observations were conducted on the sample of Co_3_O_4_ prepared with 15 mL of rotten *Solanum melongena* juice. Fig. 3 provides a summary of the results. It was observed by SEM that the sample prepared with *Solanum melongena* juice contained aggregated particles of various shapes, including small nanorods (Fig. 3a) or small angular nanoparticles (Fig. 3b), which are primarily of a few tens of nanometers in diameter within the 10-nanometer range for both nanorods and nanoparticles. As demonstrated by XRD measurements (Fig. 1), HRTEM micrographs (Fig. 3c and d) using Fast Fourier Transform (FFT) (Fig. 3e) reveal polycrystallinity. The *d*-spacing of 0.47 nm between adjacent lattices corresponds to the lattice distance of (111) crystal planes of Co_3_O_4_ phase and the FFT patterns corresponding to the lattice plane of Co_3_O_4_ (111), (220), (311), (400) are clearly identified in Fig. 3e, confirming the cubic phase structure *Fd*3̄*m* of the Co_3_O_4_ sample prepared with 15 mL of rotten *Solanum melongena* juice.

**Fig. 4:**
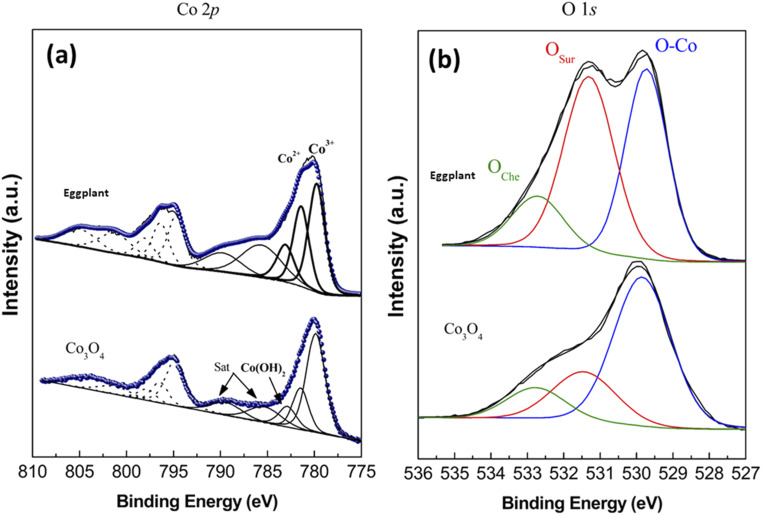
X-ray core level spectra of pure Co_3_O_4_ nanostructures and 15 mL rotten *Solanum melongena* juice mediated Co_3_O_4_ nanostructures (a) Co 2p_3/2_, (b) O 1s.

A study of the surface chemical composition was conducted by XPS and the measured spectra are shown in Fig. 4. In Fig. 4, Lorentzian–Gaussian functions fitting models were used to obtain high resolution XPS spectra for Co 2p and O 1s. Fig. 4a shows the fitted Co 2p spectra for pure Co_3_O_4_ and 15 mL rotten *Solanum melongena* juice mediated Co_3_O_4_, which demonstrate distinctive satellite shake up peaks due to Co(iii) and Co(ii). The relative % of these variable oxidation states were calculated using the corresponding Co^3+/^Co^2+^ ratio that was 2.8 and 1.4 for pure and *Solanum melongena* samples respectively, which indicates a higher amount of Co(ii) species on the surface of *Solanum melongena* plant juice mediated Co_3_O_4_. By enriching surface oxygen vacancies, Co(ii) species have been shown to be highly active for OER.^43–45^*Solanum melongena* has been found to produce a wide range of phytochemicals, which have been shown to enhance the presence of Co(ii) species on its surface, and the higher the Co(ii) content on the surface of cobalt oxide, the greater the oxygen vacancies, thus enhancing the activity of the OER.^46^ High resolution O 1s spectra of pure Co_3_O_4_ and *Solanum melongena* mediated Co_3_O_4_ were also fitted for the illustration of surface defects contributed by the presence of oxygen vacancies as shown in Fig. 4b. Three main attributions of oxygen were noticed in the O 1s spectra for the metal–oxygen bonds (O1, 529.6 eV), defective positions with oxygen vacancies (O2, 531.3 eV) and adsorbed water or organic substances (O3, 532.9 eV)^43–46^

The relative % of each oxygen contrition was found in the order O1 > O2 > O3 for pure sample and O1 ≈ O2 > O3 for *Solanum melongena* mediated sample and revealing that a greater amount of surface vacancies are present in the case of *Solanum melongena*-based sample. Quantitative information about Co(ii) and oxygen defects enabled the *Solanum melongena* mediated Co_3_O_4_ to behave as an efficient electrode material for the OER half-cell and supercapacitor applications.

**Fig. 5:**
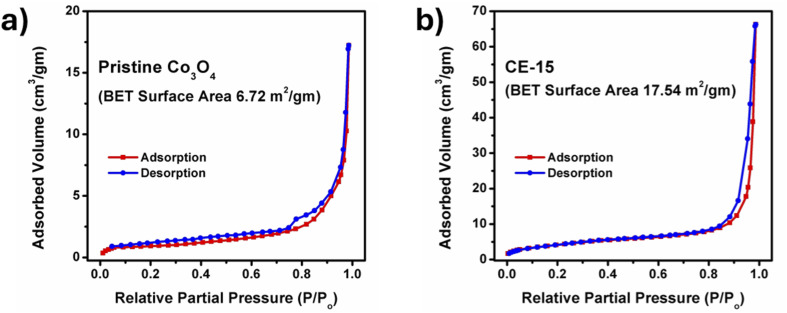
BET surface area analysis of (a) pure Co_3_O_4_ nanostructures and (b) 15 mL rotten *Solanum melongena* juice mediated Co_3_O_4_ nanostructures.

Surface area and porosity are important factors that influence the performance of nanostructured materials. We measured the specific surface area and pore-size distribution of the pristine Co_3_O_4_ and *Solanum melongena* mediated Co_3_O_4_ (CE-15) nanostructures using a nitrogen gas adsorption experiment. In Fig. 5a and b, both nanostructures produced a type-IV isotherm, indicating macro-porosity in both pristine and *Solanum melongena*-mediated Co_3_O_4_ structures. Using the multi-point BET method, the specific surface area of pristine Co_3_O_4_ was calculated. An estimated specific surface area of 6.72 m^2^ g^−1^ was observed, which is only two-fifth of the specific surface area of the *Solanum melongena* mediated Co_3_O_4_ (CE-15) nanostructure (17.54 m^2^ g^−1^). The pore volumes of pure Co_3_O_4_ and *Solanum melongena* mediated Co_3_O_4_ (CE-15) nanostructures were 1.892 × 10^−2^ cm^3^ g^−1^ and 7.106 × 10^−2^ cm^3^ g^−1^, respectively. The BET analysis has revealed that the *Solanum melongena* mediated Co_3_O_4_ (CE-15) nanostructures experienced adequate surface area and pore volume which likely enhanced the electrochemical activity^47–49^

### 3.2 Evaluation of electrochemical performance of as prepared Co_3_O_4_ nanostructures

**Fig. 6:**
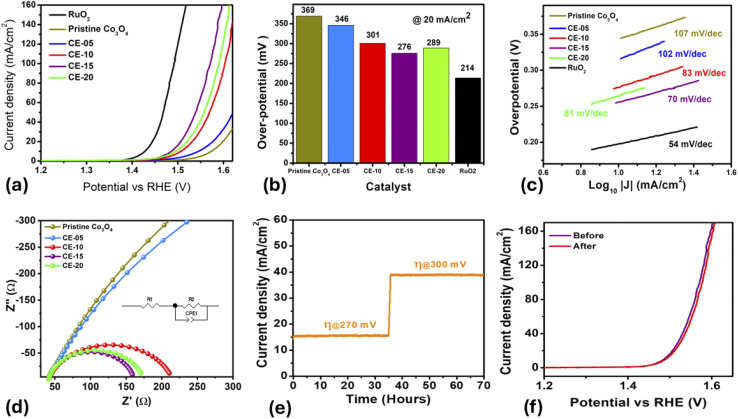
(a) OER polarization curves of pristine Co_3_O_4_ nanostructures, RuO_2_, and CE-05, CE-10, CE-15, CE-20, measured by LSV at 2 mV s^−1^ with *iR* corrected data, (b) Bar graph presentation of overpotential of each material at 20 mA cm^−2^, (c) corresponding Tafel slopes of each nanomaterial, (d) charge transfer analysis using EIS Nyquist plots of various Co_3_O_4_ nanostructures with different volumes and without rotten *Solanum melongena* juice using frequency range of 100 kHz to 0.1 Hz at an amplitude of 10 mV and OER onset potential of 1.4 *vs.* RHE, inset shows the fitted equivalent circuit in 1.0 M KOH aqueous solution, (e) durability of Co_3_O_4_ nanostructures prepared with 15 mL of rotten *Solanum melongena* for 15 hours at two different overpotentials of 270 mV and 300 mV for 70 h, (f) stability of Co_3_O_4_ nanostructures prepared with 15 mL of rotten *Solanum melongena* juice using LSV curves at 5 mV s^−1^ before and after durability test.

The electrochemical activity of Co_3_O_4_ nanostructures prepared with the use of rotten *Solanum melongena* juice was examined through various electrochemical modes such as cyclic voltammetry (CV), linear sweep voltammetry (LSV), chronopotentiometry (CP) and electrochemical impedance spectroscopy (EIS) in alkaline solution of 1 M KOH. In Fig. 6a, LSV polarization curves of pure Co_3_O_4_ and rotten *Solanum melongena* juice mediated Co_3_O_4_ nanostructures with *iR* correction. Compared with pure Co_3_O_4_ nanostructures, rotten *Solanum melongena* juice assisted Co_3_O_4_ nanostructures demonstrated superior OER performance. The improved performance of Co_3_O_4_ nanostructures could be attributed to rotten *Solanum melongena* juice's influence on shape orientation, size, and surface catalytic properties. As a result of the stabilizing, reducing, and capping properties of natural reductive and oxidative chemical compounds, the rotten *Solanum melongena* juice contributes to the variability of morphology, surface properties, and particle size. Based on the LSV analysis, we calculated the OER overpotential of rotten *Solanum melongena* juice-mediated Co_3_O_4_ nanostructures of 5 mL, 10 mL, 15 mL and 20 mL at 20 mA cm^−2^ to be 346 mV, 301 mV, 276 mV and 289 mV, respectively. However, the pure Co_3_O_4_ nanostructures experienced an overpotential of 369 mV at 20 mA cm^−2^. It has been shown in the literature that surface defects particularly the oxygen vacancies accelerate the OER kinetics and XPS analysis has demonstrated that Co_3_O_4_ nanostructures prepared with the 15 mL of *Solanum melongena* juice had significantly higher surface oxygen vacancies, which resulted in improved OER activity. In addition, the improved specific surface area of Co_3_O_4_ nanostructures prepared with 15 mL of *Solanum melongena* juice significantly improved OER performance. It has previously been demonstrated that the OER kinetics of Co_3_O_4_ nanostructures are highly influenced by the particle size and the specific area of the particles. Whereas with the decrease in particle size results in enhanced specific surface area, consequently increased OER performance has been reported.^50–55^ Interestingly, 15 mL rotten *Solanum melongena* juice assisted Co_3_O_4_ nanostructures has demonstrated low overpotential of 276 mV at 20 mA cm^−2^ for OER which is several orders lower than many of the recently Co_3_O_4_ nanostructures based electrocatalysts synthesized by different methods. As shown in Fig. 6b, a bar graph representation was made to illustrate the overpotential of as prepared Co_3_O_4_ nanostructures. Q. Xu *et al.* has designed hydrothermally Co_3_O_4−*x*_P_0.15_, prepared Co_3_O_4_ nanostructures by hydrothermal method with overpotential of 338 mV at 10 mA cm^−2^.^56^ J. Y. Xie used oil bath and annealing method for the preparation of F_0.2_-V-Co_3_O_4_-350 with overpotential of 320 mV at 10 mA cm^−2^.^57^ L. Li *et al.* has prepared 6 wt% Ni–Co_3_O_4_ and 8 wt% Ni–Co_3_O_4_ using hydrothermal method and they has shown overpotential of 330 mV and 335 mV at 10 mA cm^−2^.^58^ It was also noticed from the existing literature that the overpotential possessed by 15 mL rotten *Solanum melongena* juice mediated Co_3_O_4_ nanostructures is even lower than commercial OER electrocatalyst (IrO_2_) with overpotential of 400 mV at 10 mA cm^−2^, under the same electrolyte conditions. The OER kinetics of Co_3_O_4_ was examined through Tafel slope equation as given (*η* = *a* + *b* log *j*_0_), hereby *η* represents overpotential, *j*_0_: current density, and *b*: Tafel slope. The estimated Tafel slopes are shown in Fig. 6c. The associated Tafel slopes for 5 mL, 10 mL, 15 mL 20 mL rotten *Solanum melongena* juice mediated Co_3_O_4_ nanostructures and pure Co_3_O_4_ nanostructures were in the order 102 mV dec^−1^, 83mV dec^−1^, 70 mV dec^−1^, 81 mV dec^−1^ and 107 mV dec^−1^ respectively. The 15 mL *Solanum melongena* mediated Co_3_O_4_ nanostructures signatures the lowest Tafel slope, thus has shown effective and rapid OER kinetics. Whereas the low Tafel slope of 70 mV dec^−1^ revealed that the adsorbed intermediates are the main rate determining step for the 15 mL rotten *Solanum melongena* juice mediated Co_3_O_4_ nanostructures. This has been already generalized and described by the Krasil' Shchikov reaction model under the OER half-cell reaction in alkaline electrolytes.^59–63^

Furthermore, the electrochemical impedance spectroscopy (EIS) was performed on the 5 mL, 10 mL, 15 mL and 20 mL rotten *Solanum melongena* juice mediated Co_3_O_4_ nanostructures and pure Co_3_O_4_ nanostructures using 1.4 V *versus* RHE with sweeping frequency of 100 000 kHz to 0.1 Hz, and an amplitude of 5 mV as shown in Fig. 6d.

The charge transfer resistance (*R*_ct_) of 15 mL *Solanum melongena* mediated Co_3_O_4_ nanostructures was 119.1 Ohms which is lower than many other prepared samples including 5 mL, 10 mL and d 20 mL rotten *Solanum melongena* juice mediated Co_3_O_4_ nanostructures and pure Co_3_O_4_ nanostructures including 1498 Ohms, 172.7 Ohms, 131.8 Ohms and 2442 Ohms respectively. Hence, 15 mL *Solanum melongena* mediated Co_3_O_4_ nanostructures declared that the electrode material exhibited rapid charge transport between the electrode and electrolyte, consequently rapid OER kinetics has been demonstrated by the use of 15 mL rotten *Solanum melongena* juice.

The long-term stability of 15 mL rotten *Solanum melongena* juice mediated Co_3_O_4_ nanostructures was studied in alkaline solution of 1 M KOH for 70 hours using chronopotentiometry measurement at two different overpotentials of 270 mV and 300 mV as shown in Fig. 6e. The durability is one of the most important parameters for the nonprecious electrocatalysts in order to verify their practical aspects. The purpose of using two different current densities was to evaluate the durable behavior of rotten *Solanum melongena* juice mediated Co_3_O_4_ nanostructures in terms of variation in current density, but the observed performance of material remained unaltered. This performance evaluation indicator has confirmed that the electrode material has not brought any significant variation in the change in potential even the oxygen gas bubbling was easily produced without any effect on the removal or ineffectiveness of catalytic material. Based on these observations, it is safe to say that the proposed 15 mL rotten *Solanum melongena* juice mediated Co_3_O_4_ nanostructures could be used as an alternative and promising electrocatalyst for long term water splitting. The stability of as synthesized Co_3_O_4_ nanostructures using 15 mL of rotten *Solanum melongena* juice before and after durability was also investigated through LSV polarization curves as shown in Fig. 6f. It was seen obviously from the LSV polarization curves that Co_3_O_4_ nanostructures exhibited the same onset potential, overpotential and current density, indicating the stability of electrode material and its applicability for the practical applications. The stability of the nanostructure was further analyzed by SEM analysis after the chronopotentiometry analysis for 70 hours as shown in ESI Fig. (S1).† The morphology of the nanostructure was unaffected which confirms that CE-15 nanostructures are highly stable and material can be used for long term applications.

To support the LSV and Tafel analysis, the *C*_dl_ (electric double layer capacitance) of 5 mL, 10 mL, 15 mL and 20 mL rotten *Solanum melongena* juice mediated Co_3_O_4_ nanostructures and pure Co_3_O_4_ nanostructures were estimated. For this purpose, cyclic voltammetry curves were recorded with non-faradic regions at fixed potential range from (1 to 1.22 V) *versus* RHE as shown in Fig. (S2).† A linear relationship between difference of anodic and cathode current densities divided by 2 at various sweeping scan rates was built.^60–63^ The electrochemical active surface area (ECSA) was estimated using relations (ECSA = *C*_dl_/*C*_S_), herein *C*_S_ is specific capacitance of material.^64,65^ The 5 mL, 10 mL, 15 mL and 20 mL *Solanum melongena* mediated Co_3_O_4_ nanostructures and pure Co_3_O_4_ nanostructures exhibited the ECSA values of 3.4 μF cm^−2^, 15.1 μF cm^−2^, 19.4 μF cm^−2^, 17.3 μF cm^−2^ and 1.6 μF cm^−2^ respectively as shown in Fig. (S2).† The observed ECSA values again supported the obtained results of OER performance *via* low overpotential and the Tafel slope of 15 mL rotten *Solanum melongena* juice mediated Co_3_O_4_ nanostructures. The summary of OER performance of different Co_3_O_4_ nanostructures is given in ESI Table (S2).† The performance of 15 mL rotten *Solanum melongena* juice mediated Co_3_O_4_ was compared with the recently reported catalysts in terms of overpotential, electrolyte condition as given in ESI Table (S3).† From the comparative analysis, it was seen that the presented synthetic method is facile, scale up, ecofriendly and environment friendly and the proposed catalyst has superior OER activity to many of the recently reported catalysts. The rotten *Solanum melongena* is attractive and useful raw material source towards the fabrication of efficient electrocatalytic materials.

### 3.3 Capacitive evaluation of rotten *Solanum melongena* juice mediated Co_3_O_4_ nanostructures

**Fig. 7:**
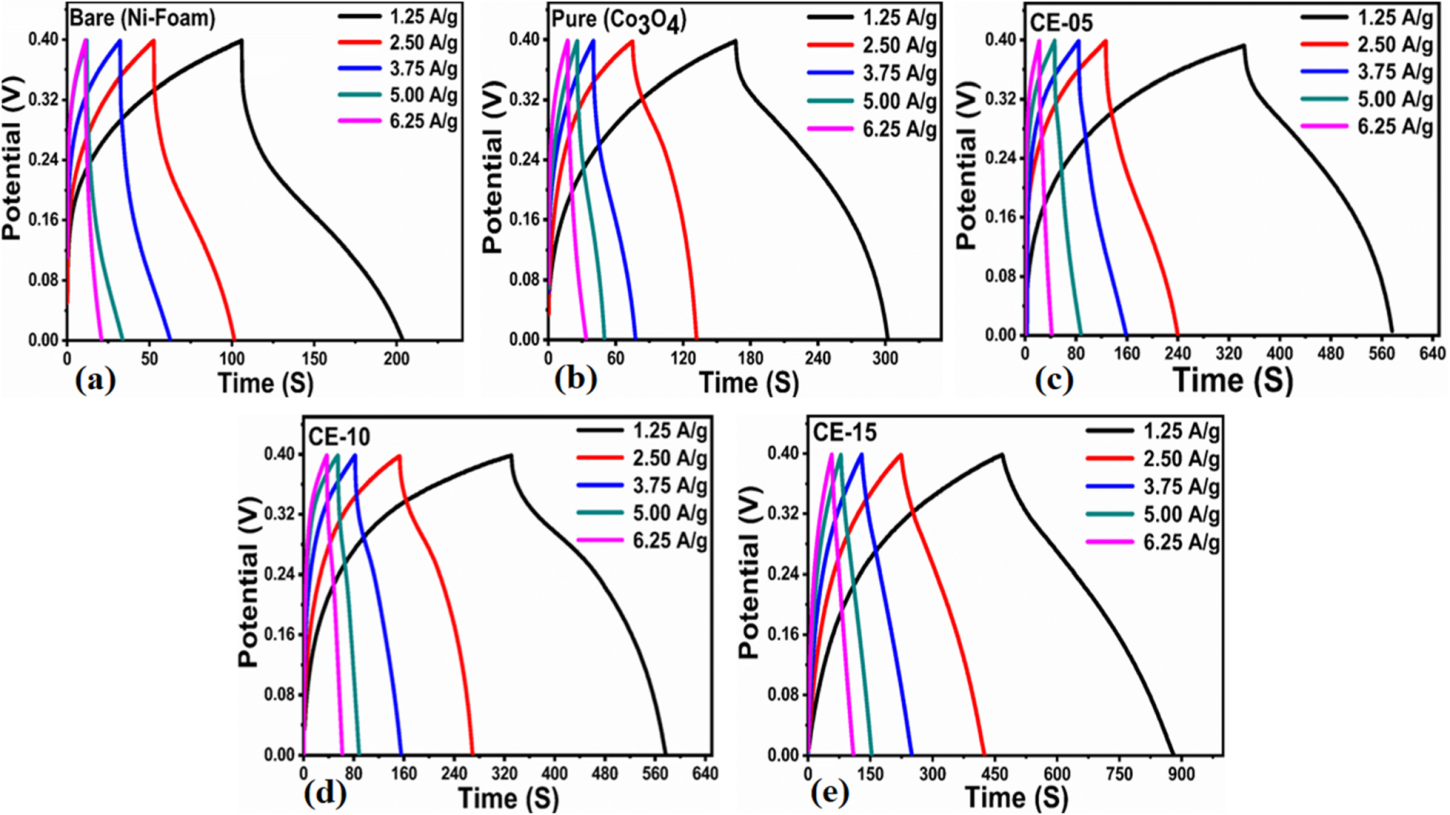
(a–e) Co_3_O_4_ nanostructures with different volumes and without rotten *Solanum melongena* juice (like CE-05, CE-10, CE-15, CE-20, CE stands for cobalt oxide-*Solanum melongena* juice) for the illustration of redox aspects using GCD curves at various current densities in 3.0 M KOH aqueous solution.

The preliminary capacitance properties of *Solanum melongena* mediated Co_3_O_4_ nanostructures were investigated through cyclic voltammetry (CV). For supercapacitor performance, a three electrodes cell set up built in 3.0 M KOH electrolytic cell solution. Hence, the analysis was performed for the exploration of capacitive properties of bare nickel foam, green mediated Co_3_O_4_ nanostructures, and Co_3_O_4_ nanostructures without the *Solanum melongena* juice. The CV curves of 5 mL, 10 mL and 15 mL rotten *Solanum melongena* juice mediated Co_3_O_4_ nanostructures and pure Co_3_O_4_ nanostructures were measured using a potential window of 0.0 to 0.5 V against (Ag/AgCl) under various sweeping scan rates such as 10, 20, 30, 40, 50 and 60 mV s^−1^ as shown in Fig. (S3a–e).† From the CV curves behavior, it is noticed that the different *Solanum melongena* mediated Co_3_O_4_ nanostructures and pure Co_3_O_4_ nanostructures under the influence of various scan rates, indicating the variable CV curve shape with sweeping potential. The CV curves at different scan rates of bare nickel foam are shown in Fig. (S3a).† It is obvious for the pure Co_3_O_4_ nanostructures that the oxidation peak potential was shifted from 0.43 V to 0.45 V, whereas the reduction potential peak was shifted from 0.31 V to 0.27 V towards higher potential in terms of oxidation potential and lower potential in terms of reduction potential as shown in Fig. (S3b).† While for the Co_3_O_4_ nanostructures synthesized with 5 mL of *Solanum melongena* juice has revealed shift in oxidation peak from 0.36 V to 0.39 V at increasing scan rate, whereas the reduction potential was shifted from 0.3 V to 0.28 V at increasing scan rate as shown in Fig. (S3c).† The CV curves was also recorded for the 10 mL of *Solanum melongena* juice mediated Co_3_O_4_ nanostructures at various scan rates and the corresponding peak shift in potential for oxidation was notice from 0.39 V to 0.41 V, and the reduction peak potential shift was observed from 0.27 V to 0.21 V with increasing scan rate as shown in Fig. (S3d).† The CV curves ate different scan rates were measured for the 15 mL rotten *Solanum melongena* juice mediated Co_3_O_4_ nanostructures and the shift in oxidation potential from 0.35 V to 0.38 V, however for the reduction potential peak shift was found from 0.3 V to 0.27 V with increasing scan rates as shown in Fig. (S3e).† In all samples, there was an increasing shift in oxidation potential with an increasing scan rate, however there was decrease shift in reduction potential with increasing scan rate. This is because of the increased internal resistance and a polarization effect caused with increasing scan rate.^66^ Moreover, the sharp and wide redox pair of peaks have been noticed at the nonlinear region of CV curves, revealing the pseudo-capacitance characteristics of the different *Solanum melongena* mediated Co_3_O_4_ nanostructures. Both the anode and cathode peaks were clearly shown. The redox peaks were very sharp and homogenous with increasing scan rate from 10 mV s^−1^ to 60 mV s^−1^, suggesting the favorable reversible redox behaviors.^67^ The Co_3_O_4_ nanostructures were describing the electron or charge transfer through CV analysis in 3.0 M electrolytic solution of KOH. The redox behavior of CV curves at different scan rates for each material was characterized with the electrochemical double layer capacitance (EDLC). The previous CV results on the Co_3_O_4_ nanostructures illustrated the reaction mechanisms in various electrolytes are fully justifying the presented CV results. The Dunn method was used to evaluate the diffusion and capacitive presentation through CV curves and the distribution of diffusion and capacitive at 60 mV s^−1^ is shown in Fig. (S4).† It could be seen that the capacitive percentage of was successively increased with increasing amount of *Solanum melongena* used during the synthesis of Co_3_O_4_ nanostructures. The diffusion and capacitive plots were illustrated using CV curves at different scan rates as shown in Fig. S5.† It was found that the 15 mL *Solanum melongena* assisted Co_3_O_4_ nanostructures resulted larger capacitive behavior than the 5 mL and 10 mL *Solanum melongena* mediated Co_3_O_4_ nanostructures and pure Co_3_O_4_ nanostructures. The features of CV curves are highly depending on the shape orientation, surface, size and structural properties of as prepared Co_3_O_4_ nanostructures. Based on these aspects from CV analysis, it is safe to say that the Co_3_O_4_ nanostructures have high potential and promising activity towards pseudo capacitance applications.^68,69^ The galvanic charge–discharge (GCD) curves were recorded for bare nickel foam, *Solanum melongena* mediated Co_3_O_4_ nanostructures and pure Co_3_O_4_ nanostructures using various current densities such as 1.25 A g^−1^, 2.5 A g^−1^, 3.75 A g^−1^, 5 A g^−1^ and 6.25 A g^−1^ in the potential window 0–0.45 V as enclosed in Fig. 7a–e.

The values of specific capacitance, energy density and power density were estimated through the relation:4
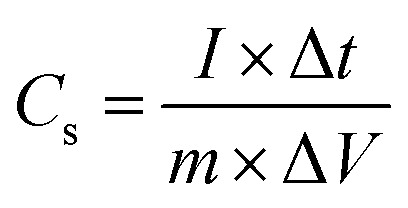
5
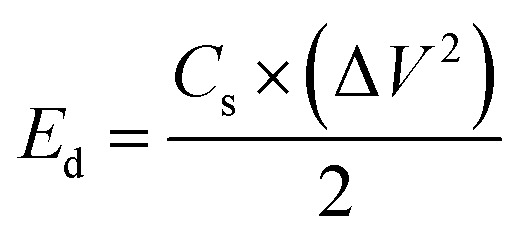
6
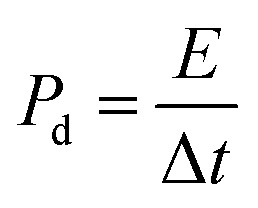
Here *C*_s_ (F g^−1^) is specific capacitance, *I* (A) current observed at the time of discharge, Δ*t* discharge time, Δ*V* voltage range and *m* (g) is active mass of electrode material.

**Fig. 8:**
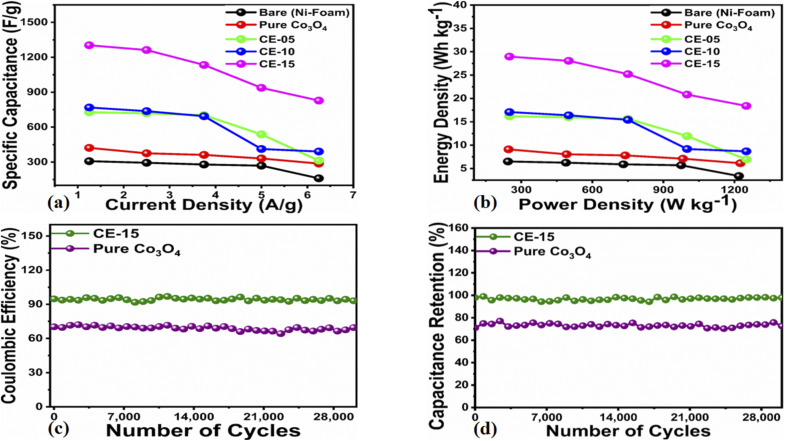
(a) Estimated specific capacitance of Co_3_O_4_ nanostructures with different volumes and without rotten *Solanum melongena* juice (like CE-05, CE-10, CE-15, CE-20, CE stands for cobalt oxide-*Solanum melongena*) from GCD curves in 3.0 M KOH aqueous solution, (b) related energy density of Co_3_O_4_ nanostructures with different volumes and without *Solanum melongena* for the illustration of redox aspects (c) coulombic efficiency of Co_3_O_4_ nanostructures with different volumes and without rotten *Solanum melongena* juice for the coulombic efficiency (d) corresponding percentage retention specific capacitance of Co_3_O_4_ nanostructures with different volumes and without rotten *Solanum melongena* juice from 30 000 GCD repeatable cycles in 3.0 M KOH for the demonstration of cycling stability.

From the GCD curves, it was noticed that they are associated with the pair of platforms, describing its pseudocapacitive properties and typical redox process took place at the time of charge–discharge phenomenon. Eqn (4) was used to estimate the specific capacitance values for each electrode materials and they are given in Table 2. It could be noticed that the 15 mL rotten *Solanum melongena* juice mediated Co_3_O_4_ nanostructures experienced the highest amount of specific capacitance 1303.13 F g^−1^ at 1.25 A g^−1^. Hence, the GCD profiles as synthesized *Solanum melongena* mediated Co_3_O_4_ nanostructures suggest that migration and diffusions of ions through 3.0 M KOH electrolytic solution has significantly enhanced the electrochemical capacitance as shown in Fig. 8a. While the *Solanum melongena* juice mediated Co_3_O_4_ nanostructures were exhibiting the specific capacitance of 421.95 F g^−1^ at 1.25 A g^−1^ and the bare nickel foam had specific capacitance of 307.69 F g^−1^ at 1.25 A g^−1^. The enhancement in the specific capacitance magnitude is highly dependent on the electrical conductivity, redox properties, and dimension of nanoparticles tailored by the *Solanum melongena* juice *via* the presence of green reducing, capping and stabilizing agents.^70–73^ For other electrode materials such as 5 mL and 10 mL *Solanum melongena* mediated Co_3_O_4_ nanostructures possessed the specific capacitance values of 728.13 F g^−1^ at 1.25 A g^−1^ and 768.75 F g^−1^ at 1.25 A g^−1^ respectively. While the estimated values of specific capacitance at different higher current densities are given in Table 2. Moreover, the observable specific capacitance values depend on the surface area of electrode material, shape orientation and variability in electrolyte concentration. Interestingly, it has been found that the synthetic method used for the development of electrode materials is also influencing on the values of specific capacitance.^74–76^ The comparative analysis was also performed of presented electrode material with the recently published works on the electrode materials for supercapacitor applications as given in ESI Table (S4).† The performance evaluation suggested that the proposed electrode material has equal or superior pseudo capacitance performance to many of the recently reported electrode materials in terms of facile synthesis, low cost, high specific capacitance, high power and energy density. The estimated power density, energy density from GCD profiles of different materials and specific capacitance retention % and coulombic efficiency % are shown in Fig. 8b. The energy density of pure Co_3_O_4_ nanostructures, bare nickel foam, 5 mL, 10 mL and 15 mL rotten *Solanum melongena* juice mediated Co_3_O_4_ nanostructures was estimated in the order 9.10, 6.50, 17.08, 16.18 and 28.96 W h kg^−1^ respectively. While the coulombic efficiency and specific capacitance retention % for pure Co_3_O_4_ nanostructures was observed as 64–72% and 70–77% for 30 000 repeatable GCD cycles at 1.25 A g^−1^ respectively as shown in Fig. 8c. However, the 15 mL *Solanum melongena* mediated Co_3_O_4_ nanostructures exhibited highly enhanced coulombic efficiency and specific capacitance retention % as 91–97% and 94–99% for 30 000 repeatable GCD cycles respectively as shown in Fig. 8d. These obtained results of cyclic stability are better in cycling stability to many of the recent pseudo capacitance electrode materials.^77–80^ Moreover, the rotten *Solanum melongena* juice has enhanced the capacitance of Co_3_O_4_ nanostructures but the coulombic efficiency was slightly deteriorated because of several factors such internal resistance, poor ionic conductivity and IR drop.^81^ From the long cyclic stability for the maintenance of specific capacitance revealed that the green reducing, capping and stabilizing agents from rotten *Solanum melongena* juice has drastically played a vital to strengthen the electrochemical capacitance performance of Co_3_O_4_ nanostructures. Hence, we have seen the formation of Co_3_O_4_ nanostructures by the use of *Solanum melongena* juice and having relatively smaller size and exhibited high surface area. For designing the efficient electrochemical supercapacitors, the large surface areas of active electrode is required due to high adsorption of hydroxide ions from the electrolytic solution, hence high charge storage capacity is expected. The large surface area of Co_3_O_4_ nanostructures obtained through the use of 15 mL of *Solanum melongena* juice and offered the frequent interaction with the hydroxide ions and relatively higher adsorption rate was found. Hence, the enhanced electrochemical supercapacitor performance was noticed for the Co_3_O_4_ nanostructures prepared with the use of 15 mL of *Solanum melongena* juic. The OER and supercapacitor performances of rotten *Solanum melongena* juice assisted Co_3_O_4_ have been found superior in terms of lower overpotential than aloe vera assisted CuO and Co_3_O_4_ composite^82^ and higher specific capacitance than the milky sap of *Calotropis procera* mediated Co_3_O_4_.^83^ These favorable aspects of rotten *Solanum melongena* juice towards improving the electrochemical properties of Co_3_O_4_ are highlighting its importance for the development of next generation for electrode materials. For simplicity, the summary of observed capacitance results is enclosed in Table 2.

**Table 2:** Supercapacitor results of different prepared Co_3_O_4_ nanostructures with and without rotten *Solanum melongena* juice

Samples	Current density (A g^−1^)	Specific capacitance (F g^−1^)	Energy density (W h kg^−1^)	Power density (W kg^−1^)	Columbic efficiency (%)	Capacitance retention (%)
CE-05	1.25	728.13	16.18	250.00	—	—
	2.5	718.75	15.97	500.00		
	3.75	703.13	15.63	750.00		
	5	537.50	11.94	1000.00		
	6.25	312.50	6.94	1250.00		
CE-10	1.25	768.75	17.08	250.00	91–97%	94–99%
	2.5	737.50	16.39	500.00		
	3.75	693.75	15.42	750.00		
	5	412.50	9.17	1000.00		
	6.25	390.63	8.68	1250.00		
CE-15	1.25	1303.13	28.96	250.00	91–97%	94–99%
	2.5	1262.50	28.06	500.00		
	3.75	1134.38	25.21	750.00		
	5	937.50	20.83	1000.00		
	6.25	828.13	18.40	1250.00		
Pure (Co_3_O_4_)	1.25	421.95	9.10	246.25	64–72%	70–77%
	2.5	375.32	8.05	491.25		
	3.75	361.68	7.80	738.75		
	5	330.79	7.10	982.50		
	6.25	286.99	6.13	1225.00		
Ni-foam (bare)	1.25	307.69	6.50	243.75	—	—
	2.5	294.12	6.25	488.75		
	3.75	278.85	5.89	731.25		
	5	269.23	5.69	975.00		
	6.25	160.26	3.39	1218.75		

## 4 Conclusions

In summary, rotten *Solanum melongena* juice was evaluated for its impact on a number of structural, morphological, and chemical properties of Co_3_O_4_ nanostructures as well as its ability to enhance electrochemical performance. The Co_3_O_4_ nanostructures were prepared using 5 mL, 10 mL, 10 mL and 15 mL of rotten *Solanum melongena* juice. In particular, the 15 mL rotten *Solanum melongena* juice mediated Co_3_O_4_ nanostructures displayed well oriented morphology, excellent crystalline properties, an abundance of surface oxygen vacancies, high specific surface area, a high ECSA, and low charge transfer resistance. It has been demonstrated that these Co_3_O_4_ nanostructures have enhanced electrochemical performance towards OER with a low overpotential of 276 mV at 20 mA cm^−2^ and a Tafel slope of 70 mV dec^−1^. The rotten *Solanum melongena* juice mediated Co_3_O_4_ nanostructures were found highly durable and stable for 70 h. The Co_3_O_4_ nanostructures prepared with 15 mL of rotten *Solanum melongena* juice exhibited efficient electrode material properties for the development of an electrochemical supercapacitor and had shown a specific capacitance of 1303.13 F g^−1^ and energy density of 28.96 W h kg^−1^ at the highest power density of 250 W Kg^−1^ at 1.25 A g^−1^. The electrode material has shown an excellent percentage specific capacitance retention up to 97% for repeatable 30 000 GCD cycles at 1.25 A g^−1^. The electrode material based on Co_3_O_4_ nanostructures synthesized with 15 mL of rotten *Solanum melongena* juice possessed potential cyclic stability. Therefore, it could be used an alternative electrode material for long-term applications. These benefitting aspects of as-prepared Co_3_O_4_ nanostructures suggest that rotten *Solanum melongena* juice is a highly useful raw material source for reducing, capping and stabilizing agents for the preparation of next generation efficient electrode materials for electrochemical applications.

## Author contributions

Abdul Jaleel Laghari, did material synthesis and partial electrochemical tests; Umair Aftab, did supervision; Muhammad Ishaque Abro, did supervision; Aneela Tahira, did XRD analysis; Elmuez Dawi, did visualization and presentation of findings; Muhammad Ali Bhatti, did partial electrochemical tests; Antonia Infantes-Molina, did XPS analysis and wrote the draft; Melanie Emo, did TEM analysis and wrote the draft; Brigitte Vigolo, did SEM and TEM analysis and wrote the draft; Rafat M. Ibrahim, did validate the results and edited the draft; Zafar Hussain Ibupoto, did supervision and wrote the original draft of manuscript.

## Conflicts of interest

Authors declare no competing interests in the resented research work.

## Supplementary Material

RA-015-D5RA02960K-s001

## Data Availability

Data sets generated during the current study are available from the corresponding author on reasonable request.
