# Supplementary material for: Facile preparation of cobalt-oxide nanostructures with enriched cobalt(ii) ion surface using Solanum melongena for energy application
Source: RSC Adv. 2025 Sep 2;15(38):31107–21. doi: 10.1039/d5ra02960k (PMC12402886; doi:10.1039/d5ra02960k)
Supplement: RA-015-D5RA02960K-s001 [file RA-015-D5RA02960K-s001.pdf]

Supplementary Information (SI) for RSC Advances.
This journal is © The Royal Society of Chemistry 2025

Supplementary Information

Facile preparation of cobalt-oxide nanostructures with enrich Cobalt(II) ion surface using solanum-melongena for energy application

Abdul Jaleel Laghari[a], Umair Aftab[a], Muhammad Ishaque Abro[a], Aneela Tahira[f], Elmuez Dawi[g], Muhammad Ali Bhatti[e], Antonia Infantes-Molina[d], Melanie Emo[c], Brigitte Vigolo[c], Rafat M. Ibrahim[h], Zafar Hussain Ibupoto[b]

[a]*Department of Metallurgy and Materials, Mehran University of Engineering and Technology, 7680 Jamshoro, Sindh, Pakistan.*
[b]*Institute of Chemistry, University of Sindh Jamshoro, 76080, Sindh, Pakistan.*
[c]*Université de Lorraine, CNRS, IJL, F-54000 Nancy, France.*
[d]*Department of Inorganic Chemistry, Crystallography and Mineralogy, Unidad Asociada al ICP-CSIC, Faculty of Sciences, University of Malaga, Campus de Teatinos, 29071, Malaga, Spain.*
[e]*Centre for Environmental Sciences, University of Sindh Jamshoro, 76080, Sindh, Pakistan.*
[f]*Institute of Chemistry, Shah Abdul Latif University Khairpur Mirs, Sindh, Pakistan.*
[g]*College of Humanities and Sciences, department of Mathematics and Sciences, Ajman University, P.O.Box 346, Ajman, United Arab Emirates.*
[h]*Physics Department, Faculty of Science, Taibah University, Al-Madaina Al Munawarah 42353, Saudi Arabia.*

**Corresponding author(s):*

Zafar Hussain Ibupoto, PhD, Email : zaffar.ibhupoto@usindh.edu.pk;

Muhammad Ishaque Abro, PhD, Email: ishaque.abro@faculty.muet.edu.pk

Table (S1): Crystallinity index, d-Spacing and lattice parameters calculated from XRD patterns.

S. No:	Sample IDs	Crystallinity index %	d-space	% Variance in d-space	Lattice parameter	% Variance in lattice parameter
1	Pristine	80.41	2.44	---	8.09	---
2	CE-05	79.42	2.444	0.164	8.108	0.222
3	CE-10	76.88	2.449	0.369	8.14	0.618
4	CE-15	75.38	2.455	0.615	8.184	1.162
5	CE-20	75.03	2.457	0.697	8.254	2.027

Phytochemical analysis methods

The phytochemical analysis methods was performed in following manner.

1. **CARBOHYDRATES**

   . **(General Test)**

Molish's test

Add a few drops of concentrated H_2SO_4 (from the walls of the test tube) to the 2-3 ml aqueous extraction solution (a few drops of alcoholic a-naphthol). Shake well. A violet ring forms at the intersection of the two liquids.

   . **For reducing sugar)**

Fehling's test

To the 2-3 ml aqueous extraction solution, add a few drops of concentrated H2SO4 (from the test tube walls) (a few drops of alcoholic a-naphthol). Give it a good shake. At the point where the two liquids meet, a violet ring appears.

Benedict's test: Add a few drops of concentrated H2SO4 (from the test tube walls) (a few drops of alcoholic a-naphthol) to the 2-3 ml aqueous extraction solution. Shake it thoroughly. At the spot where the two liquids contact, a violet ring develops.

- **Monosaccharides**

Barfoed's test

Equal volumes of Barfoed's reagent and test solution should be combined. Boil the mixture for one to two minutes, then let it cool. Precipitations turn red.

2. **ALKALOIDS**

- **(Wagner's)**

Wagner's reagent and 1 ml of HCl were added to the 2-3 ml filtrate, and everything was well shaken. A reddish-brown precipitate's formation indicated the presence of alkaloids.

3. **FLAVONOIDS**

- **(Lead acetate Test)**

Lead acetate solution was added to the little extract. The presence of flavonoids was indicated by the formation of a yellow precipitate.

4. **PHENOLS /TANNINS**

- **(Ferric Chloride Test)**

The extract will turn deep blue-black when 5% $FeCl_3$ solution is added.

5. **TEST FOR NON-REDUCING POLY SACCHARIDES:**

- **Iodine test:**

Mix 3ml test solution and few drops of weak iodine solution. The color turns blue; it vanishes when it boils and returns when it cools.

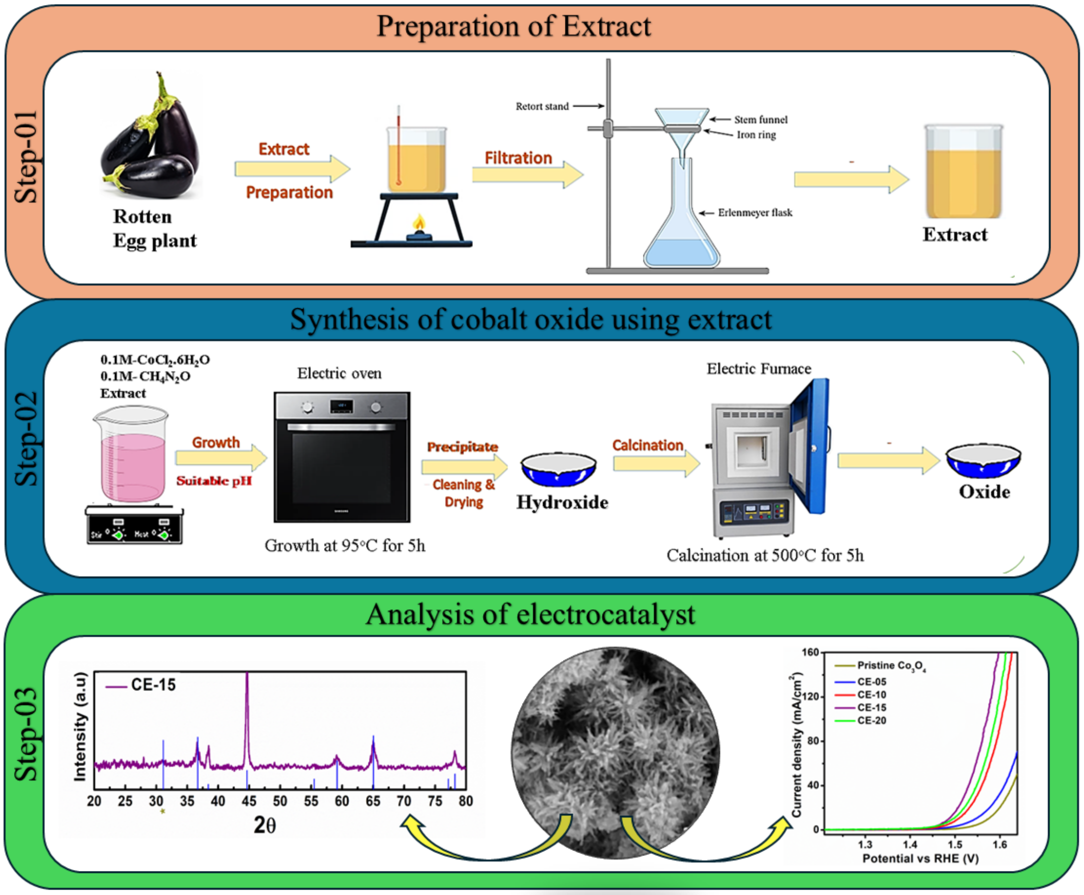

Supplementary (Scheme 1): Illustration of synthesis scheme and its interaction with heterostructure.

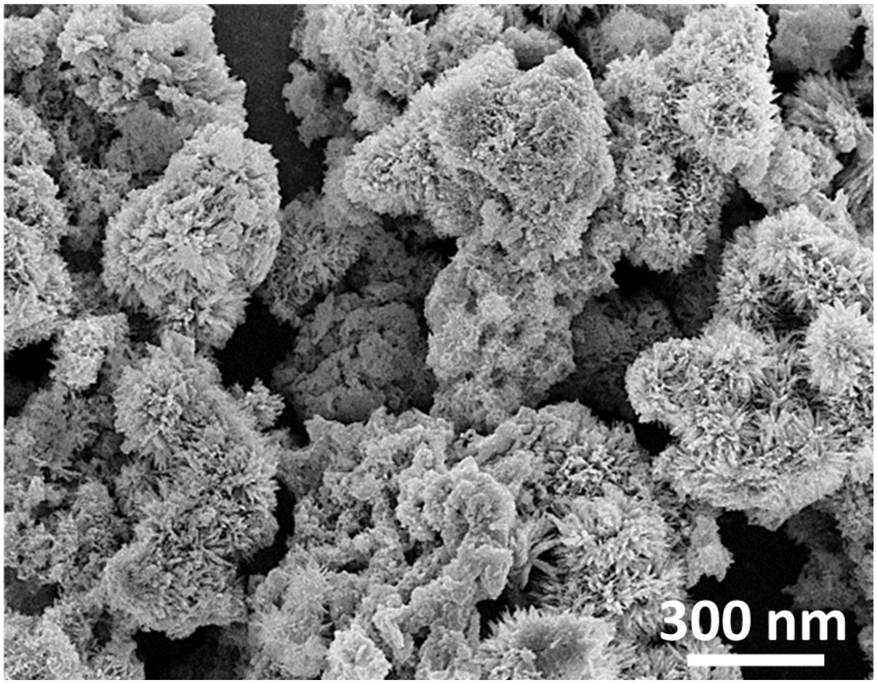

Figure (S1): Morphology after stability and durability test.

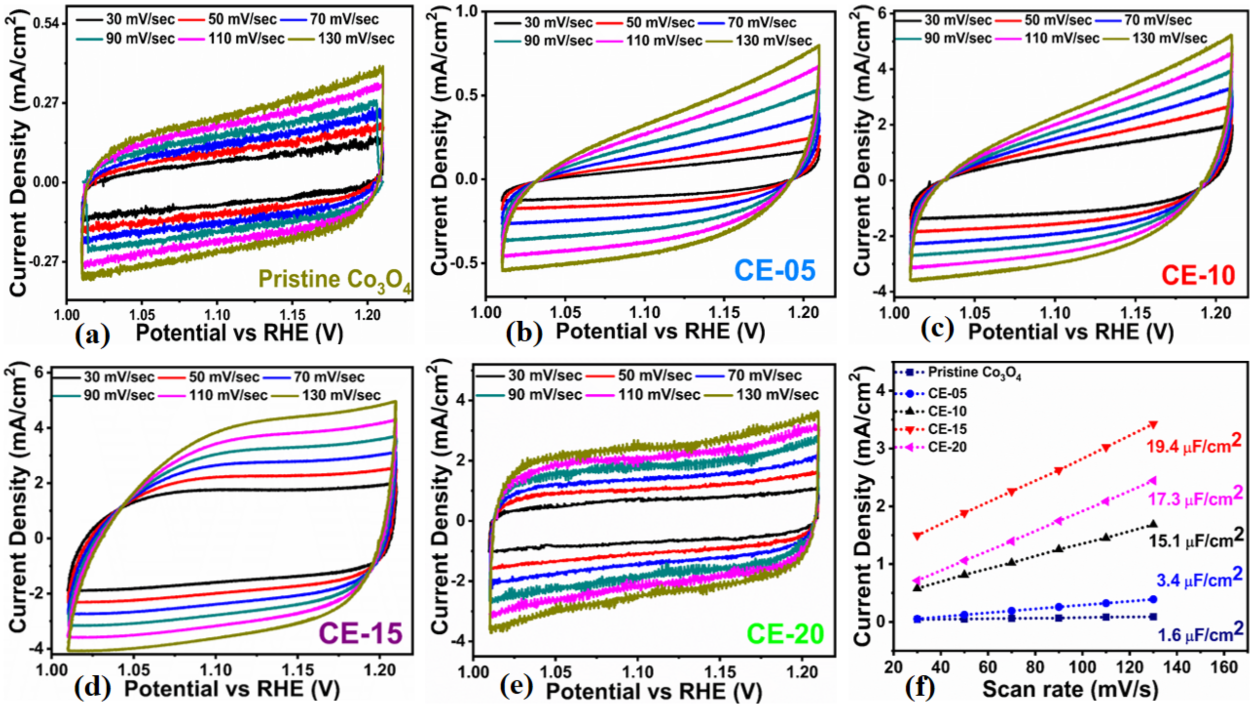

Figure (S2). (a-e) CV curves of Co_3O_4 nanostructures with different volumes and without rotten solanum melongenas juice like (CE-05, CE-10, CE-15, CE-20, CE stands for cobalt oxide) at various scan rates, (f) Related linear plot for the calculation of ECSA from the non-Faradic region of CV curves of Co_3O_4 nanostructures with different volumes and without rotten solanum melongenas juice.

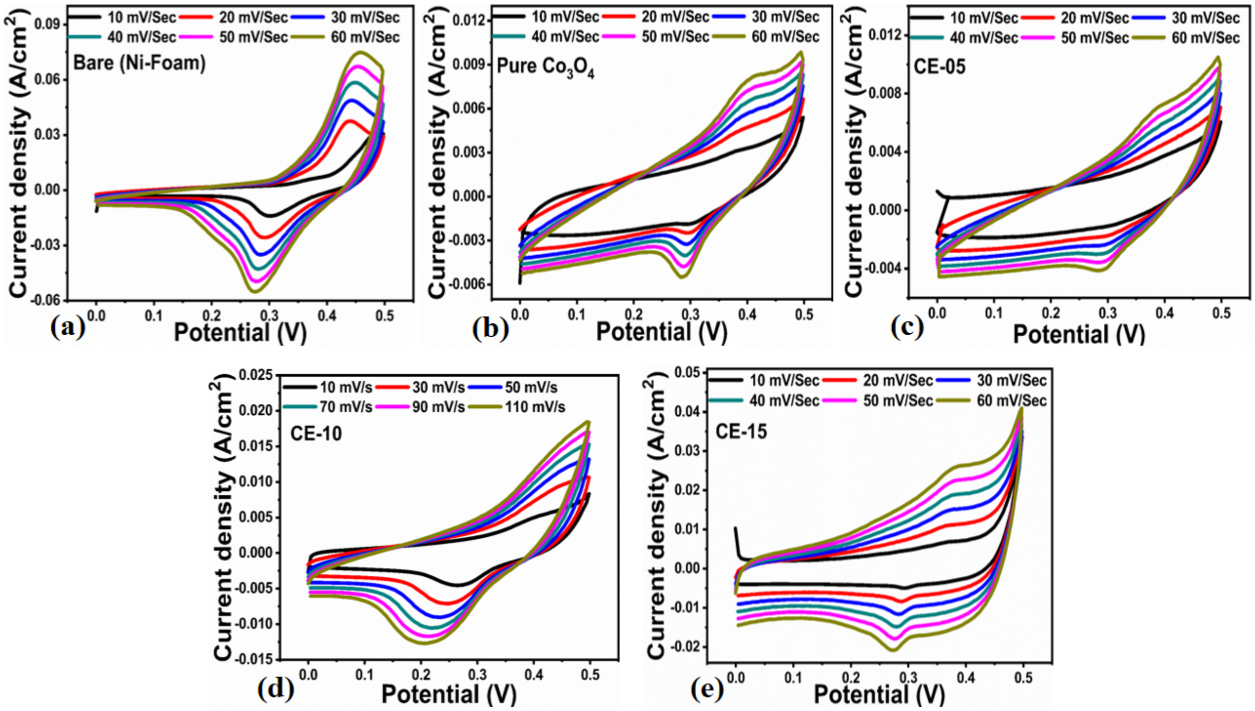

Figure. (S3): (a-e) bare nickel foam and Co_3O_4 nanostructures with different volumes and without rotten solanum melongena like (CE-05, CE-10, CE-15, CE-20, CE stands for cobalt oxide rotten solanum melongena juice) for the illustration of redox aspects using CV curves at different scan rates in 3.0M KOH aqueous solution.

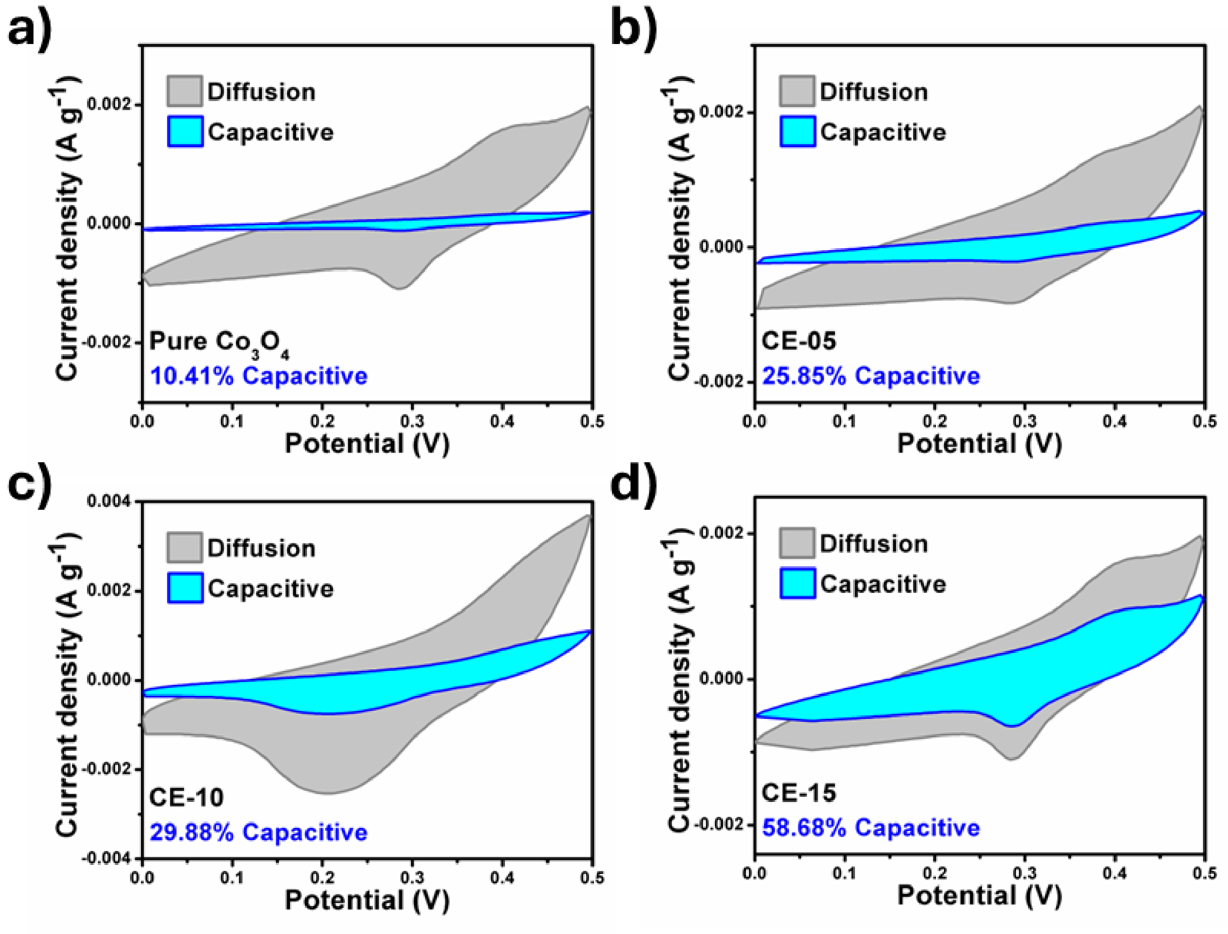

Figure (S4): (a-d) The illustration of diffusion and capacitive distribution of pure Co_3O_4 nanostructures and different (CE-05, CE-10, and CE-15 at 60 mV/s in 3M KOH solution.

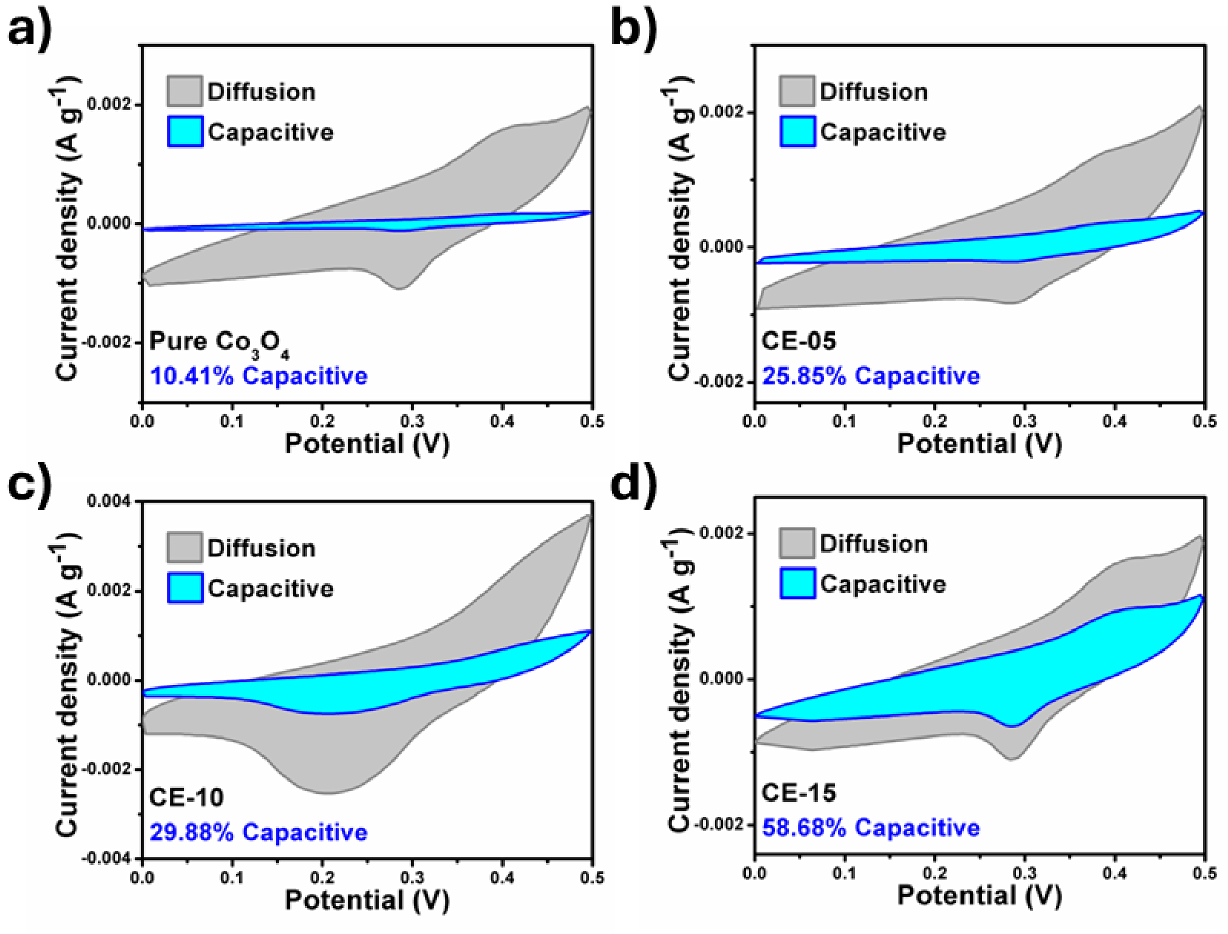

Figure (S5). (a-d) The diffusion and capacitive plots of pure Co_3O_4 nanostructures and different (CE-05, CE-10, and CE-15, CE stands for cobalt oxide rotten solanum melongenas juice) at various scan rates in 3M KOH solution .

The **specific capacitance retention (%)** is used to evaluate how much capacitance remains after repeated charge-discharge cycles, often in supercapacitor or battery testing. It is calculated using the following formula [1]:

Specific Capacitance Retention (%) $= \frac{Cn}{Co} x\ 100$ (eq. S1)

Where ,

C_0 = initial specific capacitance (1st cycle)

C_n = specific capacitance after *n* cycles

The **Coulombic Efficiency (CE)** is a measure of how efficiently charge is transferred in a battery or supercapacitor during charge/discharge cycles. It is calculated using the following formula[2]:

Coulombic Efficiency (%)= $\frac{\Delta t_{discharge}}{\Delta t_{charge}} x\ 100$ (eq. S2)

Where:

- $\Delta t_{discharge}$ = Discharging time (sec)
- Δt_{charge} = Charging time (sec)

Table (S2): Calculated values from LSV and CV curve of various Co_3O_4 nanostructures.

Catalyst	**Calculated from LSV**		**Calculated from EIS**		**Calculated from CV**	
	**Over-potential @ 20 mA cm^{-2}**	**Tafel Slope mV dec^{-1} *B***	**Charge Transfer Resistance R_{ct}**	**Double Layer Capacitance CPE_{cdl}**	**Double Layer Capacitance C_{dl}**	**Electrochemical active surface area *ECSA***
	mV	***mV/dec***	**Ω**	***mF***	***(µF cm^{-2})***	***cm^2***
Pristine Co_3O_4	369	107	1924	0.21	1.6	40
CE-05	346	102	1860	0.343	3.4	85
CE-10	301	83	599	0.40	7.2	180
CE-15	276	70	65	2.62	23	575
CE-20	289	81	122	1.67	14	350

Table (S3): Comparison of 15 mL rotten solanum melongenas juice mediated Co_3O_4 nanostructures OER performance with recently reported OER electrocatalysts.

Electrode material	Extract	Electrolyte KOH	Overpotential (mV)	Tafel (mV/dec)	References
**Co3O4 (CE-15)**	solanum melongenas	1 M	276	70	Present work
**Co3O4**	Plantago Ovata	1 M	328	71	[3]
**CuO/ Co3O4**	Aloe vera extract	1 M	453	49	[4]
**Cobalt-Based Mixed Oxide**	Tea Leaf	1 M	390	-	[5]
**Carbon doped Cobalt-oxide**	Tea leaf	1 M	380	-	[6]
**CoS/ Co3O4**	L-Lysine	1 M	304	-	[7]
**Co3O4**	Phyllanthus emblica	1 M	405	121	[8]
**Co3O4-CoO**	Gelialgas-Argagel	1 M	337	65	[9]

Table (S4): Provides comparative result of CE-15 mL rotten solanum melongenas juice mediated Co_3O_4 nanostructures with recently reported electrode materials for supercapacitor applications performance.

Material	Specific Capacitance (F/g)	Current Density (A/g)	Potential Window (V)	Energy Density (Wh/kg)	Power Density (W/kg)	Reference
**NC6**	1294 F/g	10 A/g	0.4 V	-	-	[10]
**Ni–Co–O–1**	568 F/g	20 A/g	0 to 0.5 V	19.72	5000	[11]
**NiCoF**	50 F/g	1 A/g	0 to 1 V	-	-	[12]
**NCO@MWCNT/ /MWCNT**	242 F/g	0.6 A/g	-0.5 to 2.2 V	61	2837	[13]
**$NiCo_2O_4$ (Lemon juice)**	358 F/g	0.8 A/g	0 to 0.4 V	7.96	160	[14]
**$NiCo_2O_4$ (Grapefruit)**	434 F/g	0.8 A/g	0 to 0.4 V	9.64	160	[15]
**(Co_3O_4) (MilkySap)**	699 F/g	0.8 A/g	0 to 0.4 V	30	236	[16]
**CE-15 (**solanum melongenas**)**	1303 F/g	1.25 A/g	0 to 0.4 V	28.96	250	**Present work**

References

[1] M. Sethi, U. S. Shenoy and D. K. Bhat, Nanoscale Adv., 2020, 2, 4229–4241.

[2] S. Sharma and P. Chand, Results in Chemistry, 2023, 5, 100885.

[3] R. A. Raimundo, C. S. Lourenço, N. T. Câmara, T. R. Silva, J. R. D. Santos, A. J. M. Araújo, M. M. S. Silva, J. F. G. de A. Oliveira, D. A. Macedo, U. U. Gomes, M. A. Morales and M. M. Soares, J. Electroanal. Chem., 2023, 932, 117218.

[4] D. K. Sarkar, V. Selvanathan, M. Mottakin, M. A. Islam, H. Almohamadi, N. H. Alharthi and M. Akhtaruzzaman, Int. J. Hydrogen Energy, 2024, 51, 700–712.

[5] X. Deng, C. K. Chan and H. Tüysüz, ACS Appl. Mater. Interfaces, 2016, 8, 32488–32495.

[6] A. Bähr, H. Petersen and H. Tüysüz, ChemCatChem, 2021, 13, 3824–3835.

[7] J. Hu, Z. Li, D. Zhao, Z. Han, X. Wu, J. Zhai, Z. Liu, Y. Tang and G. Fu, Green Chem., 2023, 25, 7309–7317.

[8] I. Ahmed, R. Raj, V. Burman, G. P. Singh and K. K. Haldar, Waste Biomass Valorization, 2024, DOI: 10.1007/s12649-024-02672-4.

[9] R. A. Raimundo, J. N. Silva, T. R. Silva, A. J. M. Araújo, J. F. G. A. Oliveira, L. C. de Lima, M. A. Morales, M. M. Soares and D. A. Macedo, Mater. Lett., 2023, 341, 134196.

[10] M. Kaur, P. Chand and H. Anand, J. Energy Storage, 2022, 52, 104941.

[11] H. Wang, Q. Gao and L. Jiang, Small, 2011, 7, 2454–2459.

[12] B. Bhujun, M. T. T. Tan and A. S. Shanmugam, Results Phys., 2017, 7, 345–353.

[13] M. Pathak, J. R. Jose, B. Chakraborty and C. S. Rout, J. Chem. Phys., 2020, 152.

[14] S. Kumar, A. Tahira, A. L. Bhatti, M. A. Bhatti, R. H. Mari, N. M. Shaikh, M. Y. Solangi, A. Nafady, M. Emo, B. Vigolo, A. Infantes-Molina, A. Vomiero and Z. H. Ibupoto, RSC Adv., 2023, 13, 18614–18626.

[15] S. Kumar, A. Tahira, M. Emo, B. Vigolo, A. Infantes-Molina, A. M. Alotaibi, S. F. Shaikh, A. Nafady and Z. H. Ibupoto, J. Energy Storage, 2023, 68, 107708.

[16] A. L. Bhatti, A. Tahira, S. Kumar, Z. A. Ujjan, M. A. Bhatti, S. Kumar, U. Aftab, A. Karsy, A. Nafady, A. Infantes-Molina and Z. H. Ibupoto, RSC Adv., 2023, 13, 17710–17726.
